# Electroacupuncture for post-stroke depression: a systematic review and meta-analysis of randomized controlled trials

**DOI:** 10.3389/fneur.2025.1671808

**Published:** 2025-10-17

**Authors:** Zhihui Zhang, Kaiyang Xue, Hongying Li, Mingxi Yan, Jin Cui

**Affiliations:** ^1^School of Acupuncture and Tuina, Guizhou University of Traditional Chinese Medicine, Guiyang, China; ^2^Department of Acupuncture, Tuina and Physical Therapy, The First Affiliated Hospital of Wenzhou Medical University, Wenzhou, China; ^3^Department of Acupuncture and Moxibustion, First Affiliated Hospital of Guizhou University of Traditional Chinese Medicine, Guiyang, China

**Keywords:** electroacupuncture, post-stroke, depression, meta-analysis, systematic review

## Abstract

**Background and purpose:**

Electroacupuncture (EA) is a non-pharmacological therapy within Traditional Chinese Medicine (TCM) for treating depressive disorders. This meta-analysis aims to evaluate the efficacy of EA in treating post-stroke depression (PSD).

**Methods:**

Seven electronic databases were searched up to May 31, 2025, and randomized controlled trials (RCTs) on EA for PSD were screened. After two reviewers independently screened the literature, extracted data, and assessed the risk of bias in included studies, meta-analysis was performed using RevMan software (version 5.4) and Stata/MP software (version 17.0).

**Results:**

Eleven studies involving 853 participants were included. EA significantly reduced Hamilton Depression Rating Scale (HAMD) scores (mean difference [MD] = −3.68, 95% confidence interval [CI]: −5.78 to −1.59, *p* = 0.0006). Additionally, EA significantly reduced Self-Rating Depression Scale (SDS) scores (MD = −3.08, 95% CI: −5.94 to −0.21, *p* < 0.0001) and National Institutes of Health Stroke Scale (NIHSS) scores (MD = −1.85, 95% CI: −2.93 to −0.77, *p* = 0.0008). Subgroup analysis demonstrated that EA significantly increased effective rates over simple acupuncture (RR = 1.38, 95% CI: 1.21 to 1.59, *p* < 0.0001), regional cerebral blood flow (rCBF) (MD = 24.21, 95% CI: 13.64 to 34.78, *p* < 0.0001), and plasma 5-hydroxytryptamine (5-HT) levels (MD = 16.83, 95% CI: 12.75 to 20.91, *p* < 0.0001). EA also significantly improved: stroke scores (SS) (MD = 2.56, 95% CI: 1.13 to 3.99, *p* = 0.0005), World Health Organization Quality of Life-BREF (WHOQOL-BREF) scores (MD = 1.85, 95% CI: 0.72 to 2.98, *p* < 0.0001), and Activities of Daily Living (ADL) scale scores (MD = 23.45, 95% CI: 17.38 to 29.52, *p* < 0.0001).

**Conclusion:**

EA may be an effective therapeutic approach for the comprehensive management of PSD. The observed clinical benefits of EA, including improvements in depression scale scores and quality of life metrics, may contribute to its potential utility in PSD management. However, the certainty of this evidence is limited by the low methodological quality of the available primary studies.

**Systematic review registration:**

https://www.crd.york.ac.uk/PROSPERO/search, identifier CRD420251055828.

## 1 Introduction

Stroke, a prevalent cerebrovascular disorder, affects approximately 70% of individuals in specific high-risk populations (1). It represents a leading cause of long-term disability worldwide. Post-stroke depression (PSD), one of the most prevalent and devastating neuropsychiatric complications, affects approximately 30% of stroke survivors within 5 years post-onset (2). PSD significantly impedes rehabilitation progress, compromises functional recovery, elevates mortality risk, and substantially diminishes quality of life (3). The pathogenesis of PSD involves multifactorial mechanisms, including: (1) dysregulation of monoaminergic neurotransmitter systems (serotonin, norepinephrine, dopamine); (2) neuroinflammation; (3) hypothalamic–pituitary–adrenal (HPA) axis dysfunction; and (4) structural and functional alterations in key neural circuits—particularly the prefrontal cortex, limbic system (encompassing the hippocampus and amygdala), and basal ganglia (4, 5). Although selective serotonin reuptake inhibitors (SSRIs) constitute first-line pharmacotherapy, their efficacy is often incomplete, characterized by delayed onset and adverse effects including gastrointestinal disturbances, sexual dysfunction, and potential drug interactions (6). Consequently, effective, well-tolerated, and accessible non-pharmacological interventions are urgently needed.

Acupuncture, a traditional therapeutic modality in TCM, is widely utilized in the clinical management of stroke and its complications. As a key component of complementary and alternative medicine (CAM), EA has gained international recognition for its efficacy and safety in treating neurological disorders (7). EA delivers pulsed electrical currents via specialized devices to synchronously stimulate the neuromuscular system, integrating the dual effects of traditional acupuncture and electrophysiological therapy (8). Owing to its non-pharmacological nature and absence of addiction potential, EA has been extensively applied in the prevention and treatment of diverse conditions (9). With demonstrated neuromodulatory potential and favorable safety profiles, EA has emerged as a significant CAM intervention for neuropsychiatric disorders (10). Mechanistic studies indicate that EA may ameliorate depressive symptoms through: (1) modulation of monoaminergic neurotransmission (enhancing serotonin and norepinephrine availability); (2) reduction of pro-inflammatory cytokines (e.g., IL-1β, TNF-*α*); (3) normalization of HPA axis hyperactivity (decreasing cortisol levels); (4) promotion of neuroplasticity (upregulating brain-derived neurotrophic factor [BDNF] expression); and (5) regulation of dysfunctional activity in prefrontal-limbic circuitry (11–13). Preliminary clinical trials and systematic reviews suggest promising therapeutic potential of EA for depression management across diverse populations (14). However, current studies on acupuncture for PSD exhibit limitations: (i) conflation of manual acupuncture and EA data; (ii) generalized evaluations of acupuncture therapy; consequently, failing to delineate EA-specific therapeutic mechanisms and clinical advantages (15, 16). Moreover, there is a paucity of systematic evaluations specifically examining EA’s efficacy and safety for PSD, particularly those integrating high-quality RCTs with multidimensional mechanistic exploration. Therefore, this systematic review and meta-analysis rigorously evaluates existing evidence to provide evidence-based conclusions regarding the clinical efficacy and safety of EA for PSD.

## 2 Methods

### 2.1 Study registration

This systematic review and meta-analysis was prospectively registered with PROSPERO (International Prospective Register of Systematic Reviews; registration number: CRD420251055828). The conduct and reporting adhere to the Preferred Reporting Items for Systematic Reviews and Meta-Analyses (PRISMA) 2020 statement.

### 2.2 Inclusion and exclusion criteria

Participants: Only randomized controlled trials were eligible. Patients with clinically diagnosed PSD based on validated criteria. No restrictions were applied regarding gender, age, or disease duration.

Interventions: Studies consisted of EA monotherapy or EA combined with normal treatment were included, explicitly excluding protocols incorporating antidepressant pharmacotherapy; EA parameters including acupoint selection, stimulation devices, frequency, intensity, and treatment duration were unrestricted. Control interventions comprised: (1) normal treatment, (2) simple acupuncture, or (3) other treatments to improve depressive symptoms, such as oral medication.

Outcomes: Primary outcomes were Hamilton Depression Rating Scale (HAMD) and effective rate, while secondary outcomes included: Self-Rating Depression Scale (SDS), quality of life measures (WHOQOL-BREF/ADL), National Institutes of Health Stroke Scale (NIHSS), regional cerebral blood flow (rCBF), stroke scores, plasma 5-hydroxytryptamine (5-HT) levels, and safety profiles (adverse event incidence).

Study design: Only RCTs without language restrictions were included, with exclusions applied to non-acupuncture electrical stimulation therapies, and studies lacking full-text or complete outcome data to mitigate clinical and statistical heterogeneity, respectively.

### 2.3 Search strategy

Two independent investigators (ZHZ and KYX) systematically searched seven electronic databases (CNKI, VIP, Wanfang, PubMed, Web of Science, Sinomed, Embase) from inception through May 31, 2025, using structured Boolean combinations of key terms: (“electroacupuncture” OR “electrical stimulation acupuncture” OR “holographic electroacupuncture” OR “transcutaneous acupoint electrical stimulation”) AND (“stroke” OR “cerebrovascular disorders” OR “cerebrovascular accident” OR “brain infarction” OR “ischemic stroke” OR “hemorrhagic stroke”) AND (“depression” OR “depressive disorder” OR “post-stroke depression” OR “mood disorders”). Medical Subject Headings (MeSH) terms were employed in MEDLINE/PubMed-adapted databases. Additional searches included backward citation tracking of included studies and manual searching of Google Scholar for gray literature. Taking PubMed as an example, the search query is: *(“electroacupuncture” [MeSH Terms] OR “electroacupuncture” [tiab] OR “electrical acupuncture” [tiab] OR “electrical stimulation acupuncture” [tiab] OR “transcutaneous acupoint electrical stimulation” [tiab]) AND (“stroke” [MeSH Terms] OR “stroke” [tiab] OR “cerebrovascular disorders” [MeSH Terms] OR “cerebrovascular accident” [tiab] OR “brain infarction” [MeSH Terms] OR “ischemic stroke” [tiab] OR “hemorrhagic stroke” [tiab]) AND (“depression” [MeSH Terms] OR “depression” [tiab] OR “depressive disorder” [MeSH Terms] OR “post-stroke depression” [tiab] OR “mood disorders” [MeSH Terms])*.

### 2.4 Study selection and data extraction

Two authors (ZHZ and KYX) independently screened titles and abstracts against predefined inclusion and exclusion criteria. Following initial independent screening, both reviewers examined full texts of potentially eligible studies. Data extraction was then performed independently using standardized forms. Discrepancies were resolved through consensus or adjudication by a third reviewer (HYL). Extracted data included: (1) study characteristics (first author, publication year, country); (2) participant characteristics (mean age, sample size, stroke stage); (3) intervention details; and (4) outcomes measures with adverse event reporting.

### 2.5 Data analysis

#### 2.5.1 Risk of bias assessment

Two reviewers (ZHZ and KYX) independently evaluated the risk of bias using the Cochrane Risk of Bias tool for randomized trials (RoB 2.0), covering five domains: randomization process, deviations from intended interventions, missing outcome data, outcome measurement, and selective reporting. According to the bias assessment tool, each trial was categorized as low risk, some concerns, or high risk per RoB 2.0 criteria, with overall assessments presented graphically. Disagreements were resolved through consensus discussions with a third experienced reviewer (HYL).

#### 2.5.2 Publication bias

Funnel plots were used to assess publication bias when >10 studies were included, supplemented by Egger’s statistical test to minimize limitations of visual asymmetry interpretation.

#### 2.5.3 Sensitivity analysis

Sensitivity analyses were performed by sequentially excluding studies with high risk of selection bias and those whose confidence intervals (CIs) did not overlap with the pooled effect estimate’s CI.

#### 2.5.4 Evidence certainty

Two reviewers (ZHZ and KYX) independently evaluated the certainty of evidence using the GRADE framework (Grading of Recommendations, Assessment, Development, and Evaluations). Evidence was categorized into four certainty levels: high, moderate, low, or very low, with disagreements resolved through consensus discussions involving a third experienced reviewer (HYL).

#### 2.5.5 Data synthesis strategy

Data were analyzed using Review Manager 5.4 (The Cochrane Collaboration) and Stata/MP 17.0. Dichotomous outcomes were expressed as risk ratios (RRs) with 95% confidence intervals (CIs), while continuous outcomes used mean differences (MDs) with 95% CIs. The Mantel–Haenszel (M-H) fixed-effect model was applied when *I*^2^ ≤ 50% indicated low heterogeneity; otherwise, the M-H random-effects model was employed.

#### 2.5.6 Subgroup analysis

Prespecified subgroup analyses were conducted according to intervention type, stroke stage, and baseline depression severity.

## 3 Results

### 3.1 Study selection

**Figure 1:**
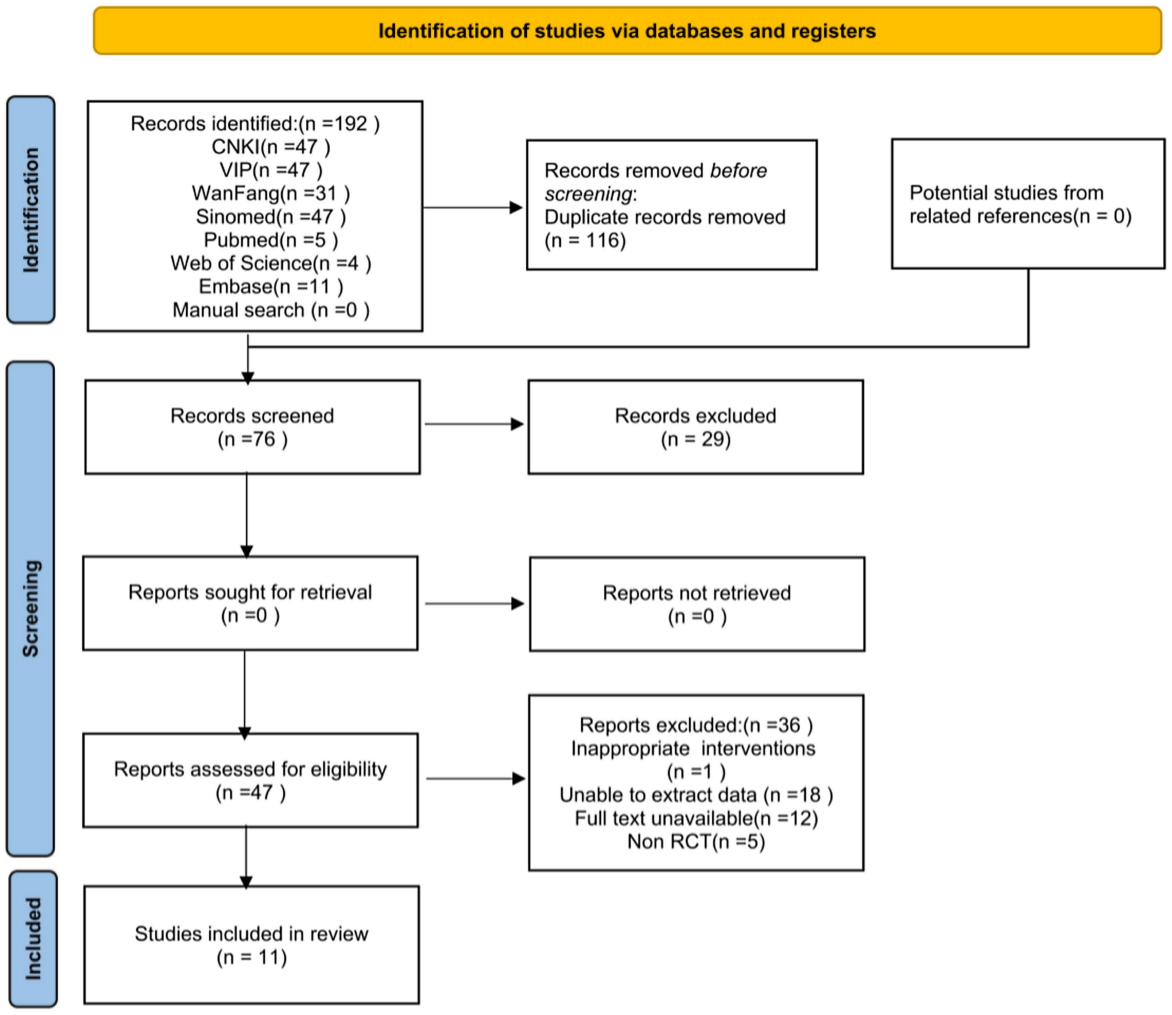
Flowchart for the identification and selection of RCTs. RCTs: random controlled trials.

The initial search identified 192 potentially relevant records, with 116 duplicates removed. Following title and abstract screening, 29 studies were excluded per eligibility criteria, leaving 47 for full-text assessment. After full-text review, 36 studies were excluded due to: intervention mismatch (*n* = 1), irretrievable data (*n* = 18), unavailable full texts (*n* = 12), or non-RCT designs (*n* = 5). Consequently, 11 studies (17–27) were included in the meta-analysis, with the selection process detailed in Figure 1.

### 3.2 Characteristics of included studies

Table 1 summarizes study characteristics. Notably, all 11 included studies were conducted in China, totaling 853 participants. Participant ages ranged from 40 to 80 years, sample sizes varied from 21 to 160, and stroke chronicity spanned 28 days to 3 years post-stroke. Three studies compared EA versus oral medication (19, 23, 25), while two examined EA plus normal treatment (NT) versus medication plus NT (21 27). One study assessed EA against simple acupuncture only (22) and another evaluated EA combined with simple acupuncture versus simple acupuncture alone (20). Additional designs included EA plus scalp acupuncture versus oral medication (26).

**Table 1:** Characteristics of included studies.

Authors, year	Country	Age (T/C)	Sample size	Stage of stroke	Type of intervention	Target outcomes
Li et al. (2015) (23)	China	62.56 ± 6.85 66.42 ± 6.25	11/10	(7.20 ± 2.35)m (7.34 ± 2.45)m	EA	1,2,7
Xian et al. (2018) (22)	China	56.93 ± 8.60 54.43 ± 8.02	30/30	(7.37 ± 2.87)m (7.07 ± 2.96)m	EA	1,2
Sang et al. (2018) (24)	China	T:56.43 ± 9.98 C:56.57 ± 8.78 C:57.63 ± 8.72	30/30/30	(6.73 ± 3.36)m (6.16 ± 4.25)m (6.29 ± 3.74)m	EA	1,2
Tang et al. (2003) (18)	China	66.97 ± 7.75 66.03 ± 7.42	30/30	(19 ± 15)m	EA + NT	1,4
Sun (2011) (26)	China	61.5 ± 11.4 62.8 ± 9.60	80/80	(6.5 ± 1.10)m (6.8 ± 0.90)m	EA	2
Zhang et al. (2013) (27)	China	43–78 44–76	34/27	(1.0−20)m (1.5−18)m	EA + NT	1,2,3
Kang et al. (2014) (21)	China	40–80	40/40	≤3 m	EA + NT	1,5,6
Huang et al. (2005) (19)	China	60.45 ± 8.42	32/31	(1.75 ± 0.06)y	EA	2,3
Dai (2009) (25)	China	42–70 46–72	30/30	(23.2 ± 3.60)d (24.6 ± 3.20)d	EA	1,2
Dong et al. (2007) (17)	China	T:58.40 ± 9.60 C:59.21 ± 7.56 C:56.61 ± 8.21	38/36/34	(2.60 ± 2.20)m (2.80 ± 2.01)m (2.50 ± 2.10)m	EA	1,2,3,8
Huang et al. (2003) (20)	China	61.96 ± 8.30	46/44	28d−38 m	EA + SA	1,2,9

d, day; m, mouth; y, year; EA, electroacupuncture; NT, normal treatment; SA, simple acupuncture; T, treatment group; C, control group, 1: Hamilton Depression Scale (HAMD), 2: Total Effective Rate, 3: Self-Rating Depression Scale (SDS), 4: World Health Organization Quality of Life Assessment-Abbreviated Version (WHOQOL-BREF), 5: National Institutes of Health Stroke Scale (NIHSS), 6: Activities of Daily Living (ADL), 7: Regional Cerebral Blood Flow (rCBF), 8:5-Hydroxytryptamine (5-HT), 9: Stroke scale.

Regarding that EA in combination with other needling techniques may improve efficacy, one study used EA plus scalp acupuncture versus no intervention (18), and two studies comparing EA against either simple acupuncture or medication (17, 24). Overall, 401 participants received EA (alone or combined with scalp acupuncture, simple acupuncture, or NT), while 452 controls received simple acupuncture only, NT, or medication. Key characteristics of included studies including country, participant age, sample size, and stroke chronicity are summarized in Table 1.

### 3.3 Risk of bias assessment

**Figure 2:**
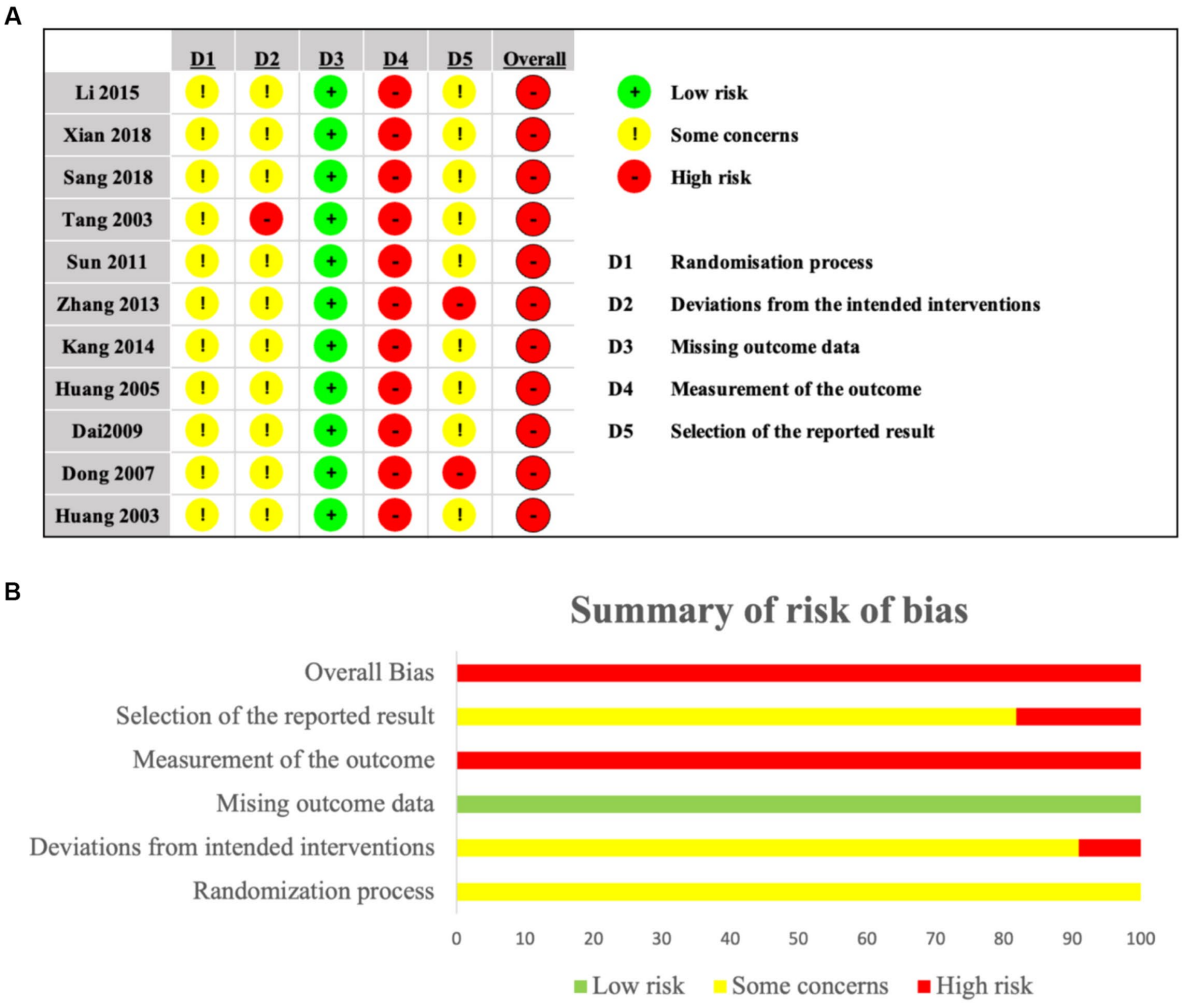
**(a,b)** Risk of bias for included studies.

The Cochrane RoB 2.0 tool assessed methodological quality across 11 included studies. Randomization concerns emerged: three studies used admission order (21, 25, 26), two employed random number generation (17, 24), one utilized minimization software (23), and five lacked methodology details (18–20, 22, 27). Consequently, all studies raised some concerns regarding allocation concealment. One study deviated from protocol-specified interventions (18), suggesting possible performance bias. While all studies demonstrated low attrition bias, all exhibited high risk in measurement bias due to unblinded outcome assessment. Selective reporting bias concerned most studies through inadequate statistical protocol documentation; two studies (17, 27), showed high risk from inconsistent depression measurement scales. Overall, studies demonstrated high risk predominantly from randomization deficiencies, intervention deviations, and outcome measurement limitations (Figures 2a,b).

### 3.4 Primary outcomes

#### 3.4.1 Outcome: HAMD

**Figure 3:**
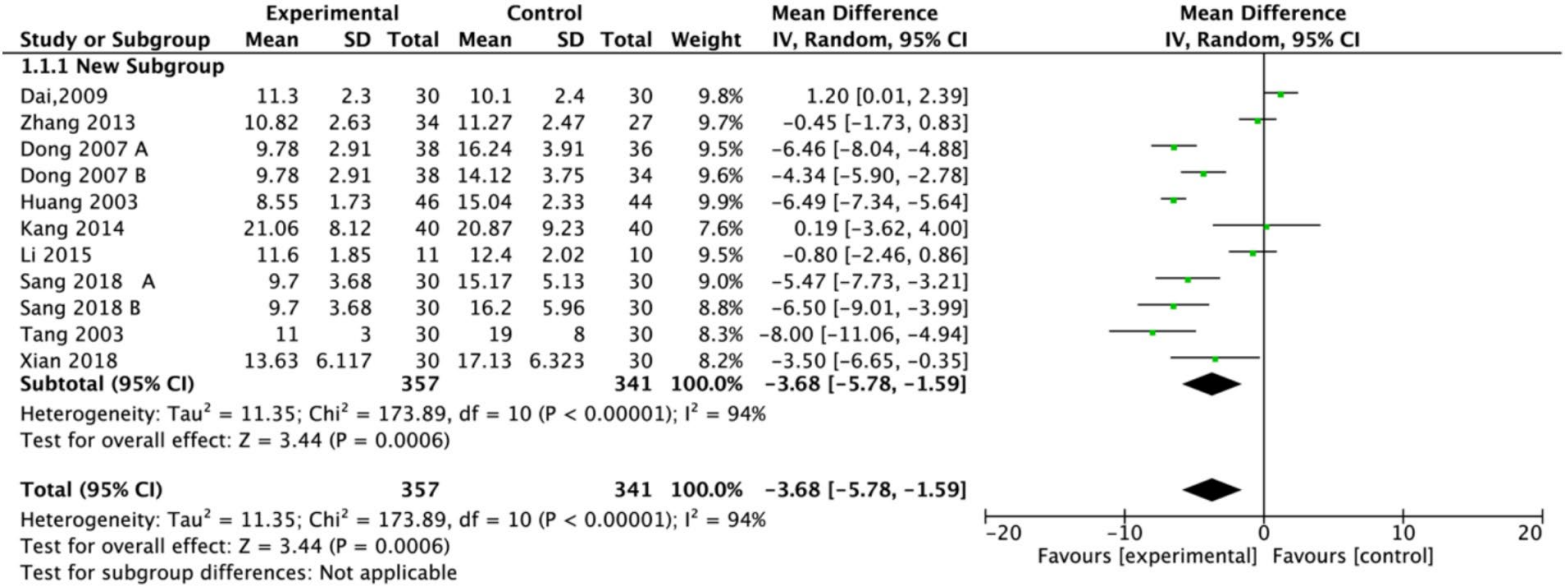
Forest plot of Hamilton Depression Scale (HAMD). Electroacupuncture vs. normal treatment or simple acupuncture. EA, electroacupuncture; SD, standard deviation; IV, inverse variance; CI, confidence interval.

The Hamilton Depression Rating Scale (HAMD) score is crucial for diagnosing and treating PSD, with lower scores indicating better treatment efficacy and improved depressive status. In this meta-analysis, nine studies assessed HAMD (17, 18, 20–25, 27), involving a total of 289 participants in the experimental groups and 341 participants in the control groups. Among these, seven studies employed a single control group design, while two utilized a dual control group design. To comprehensively analyze the data, the study splitting approach was applied to the dual control group studies, dividing each into two independent sub-comparisons (experimental group vs. control group A; experimental group vs. control group B). Due to substantial heterogeneity (*I^2^* = 94%), a random-effects model was applied. The pooled effect size (Figure 3) demonstrated a significant effect of electroacupuncture in reducing HAMD scores for PSD compared to control interventions (MD = −3.68, 95% CI: −5.78 to −1.59, *p* = 0.0006).

**Figure 4:**
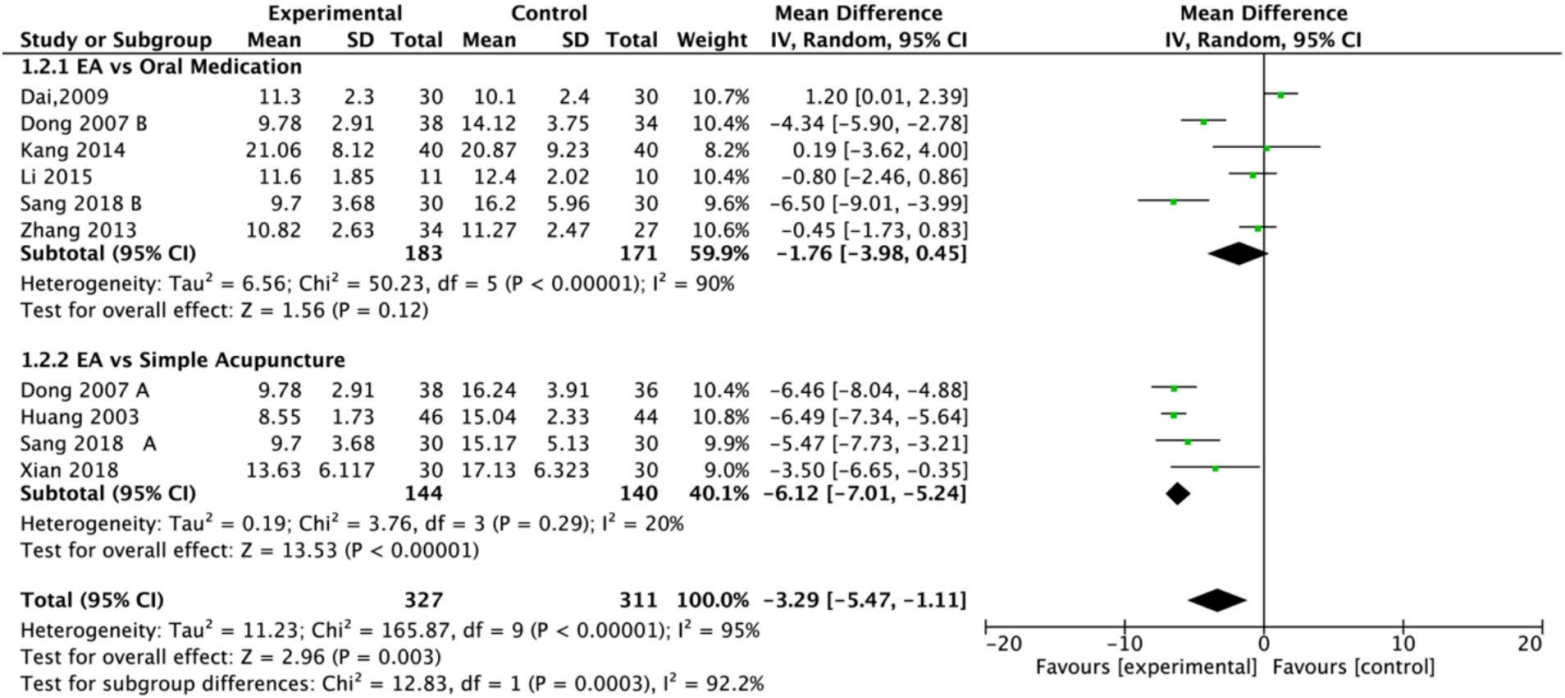
Forest plot of Hamilton Depression Scale (HAMD) including studies with subgroups. Electroacupuncture vs. oral medication and electroacupuncture vs. simple acupuncture. EA, electroacupuncture; SD, standard deviation; IV, inverse variance; CI, confidence interval.

**Figure 5:**
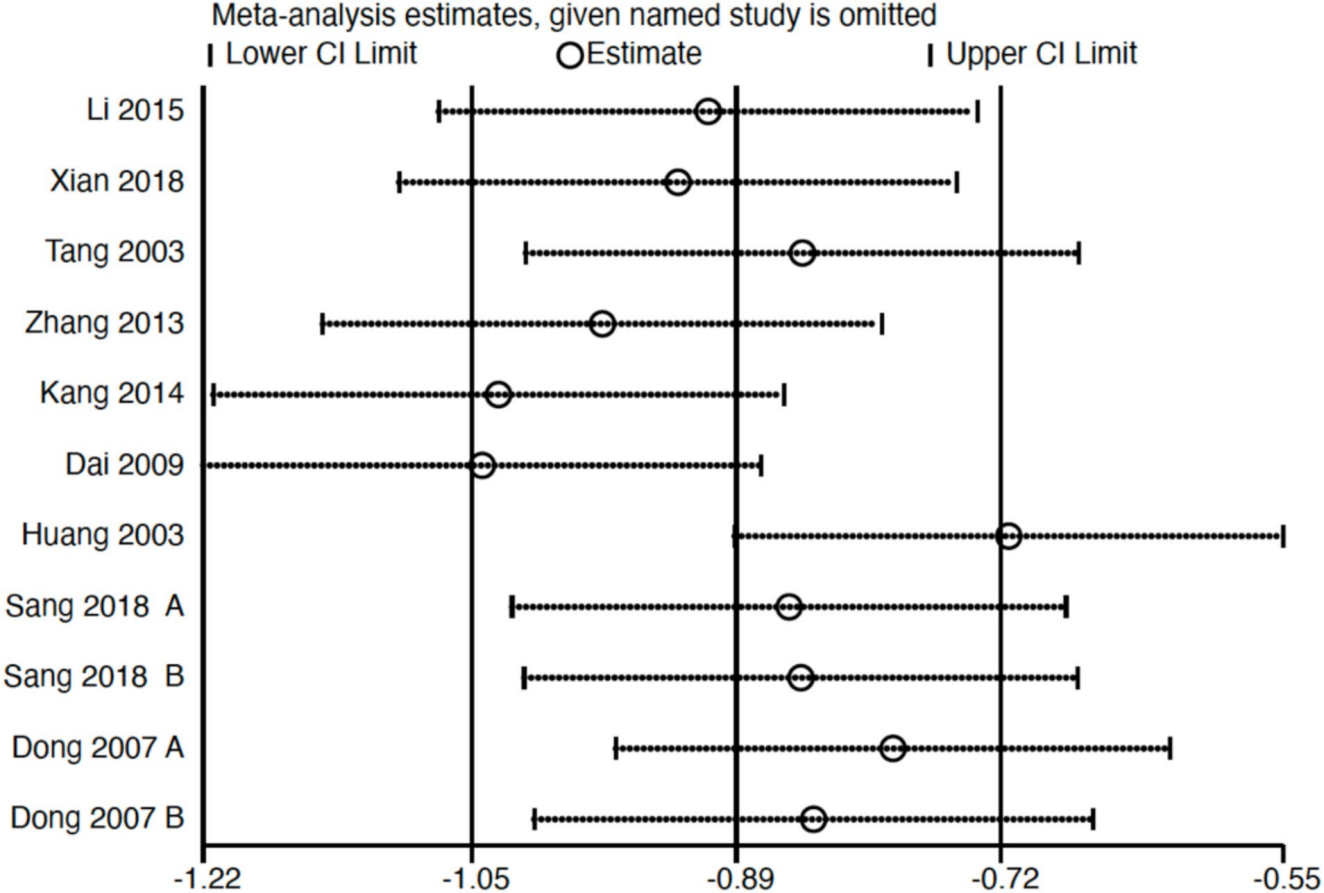
Sensitivity analysis of Hamilton Depression Scale (HAMD). CI, confidence interval.

**Figure 6:**
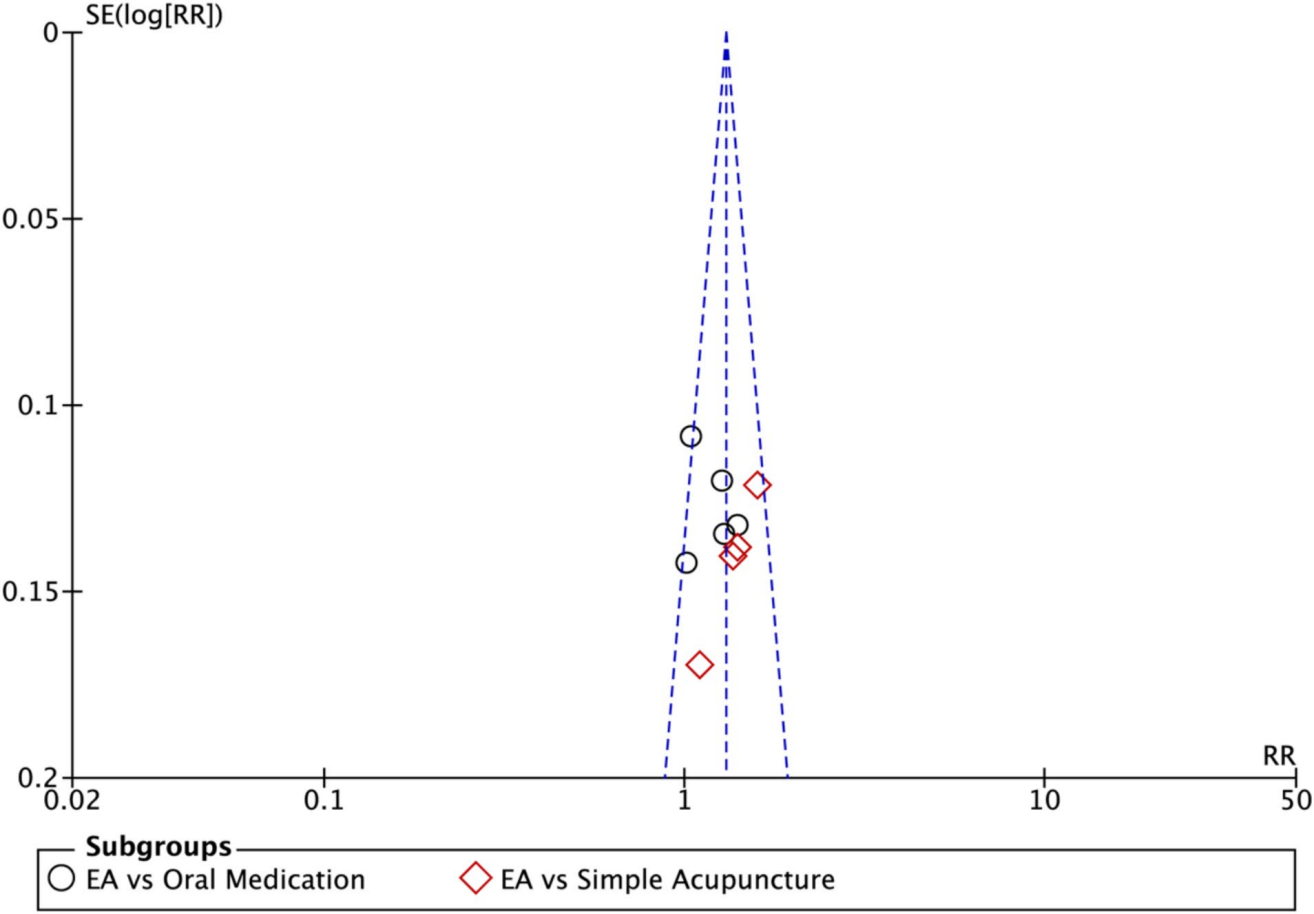
Funnel plot of Hamilton Depression Scale (HAMD). EA, electroacupuncture; RR, risk ratio; log [RR], logarithm of risk ratio.

**Figure 7:**
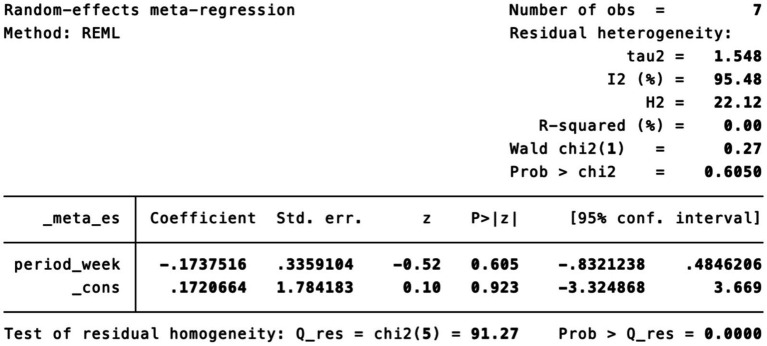
Meta-regression of treatment duration.

**Figure 8:**
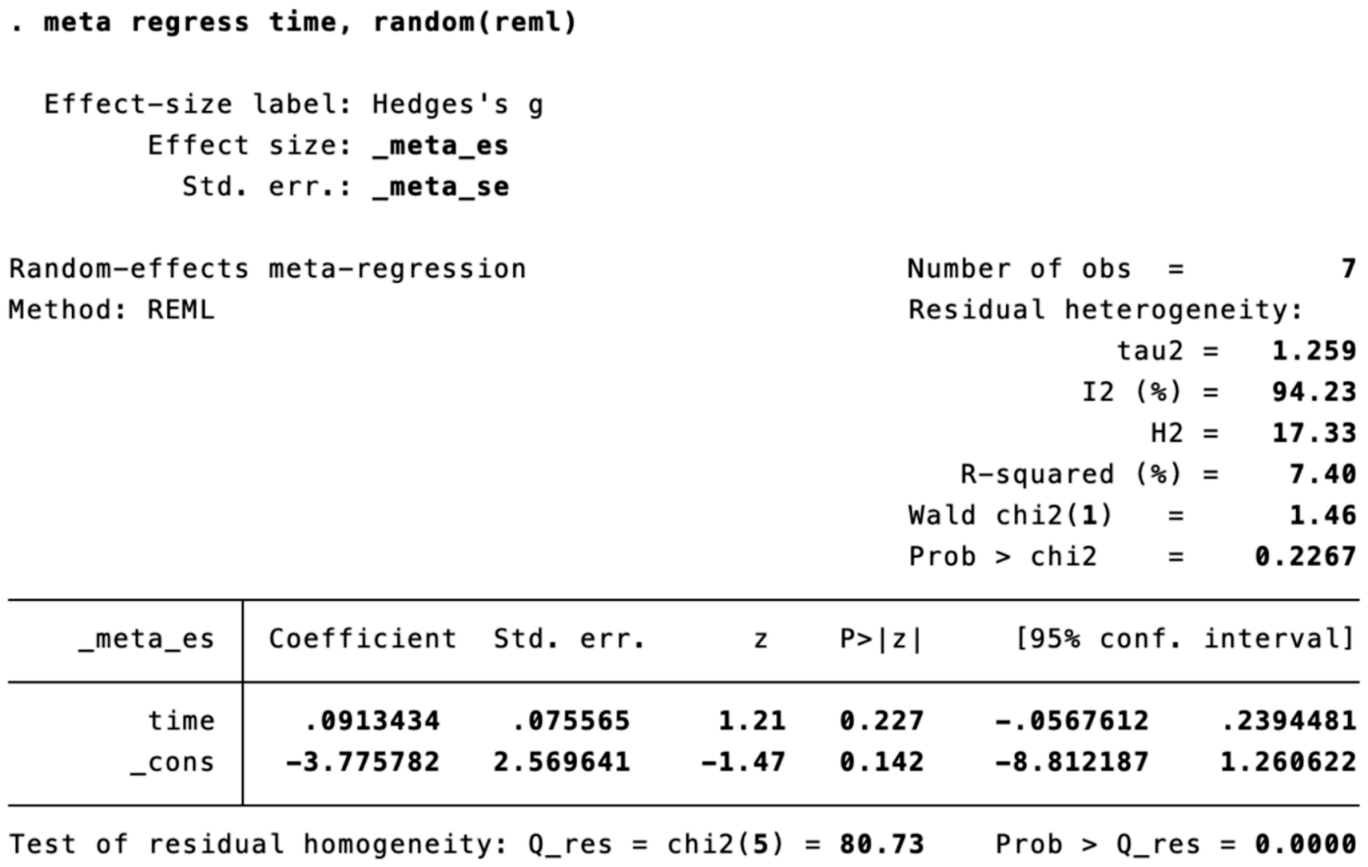
Meta-regression of treatment session length.

**Figure 9:**
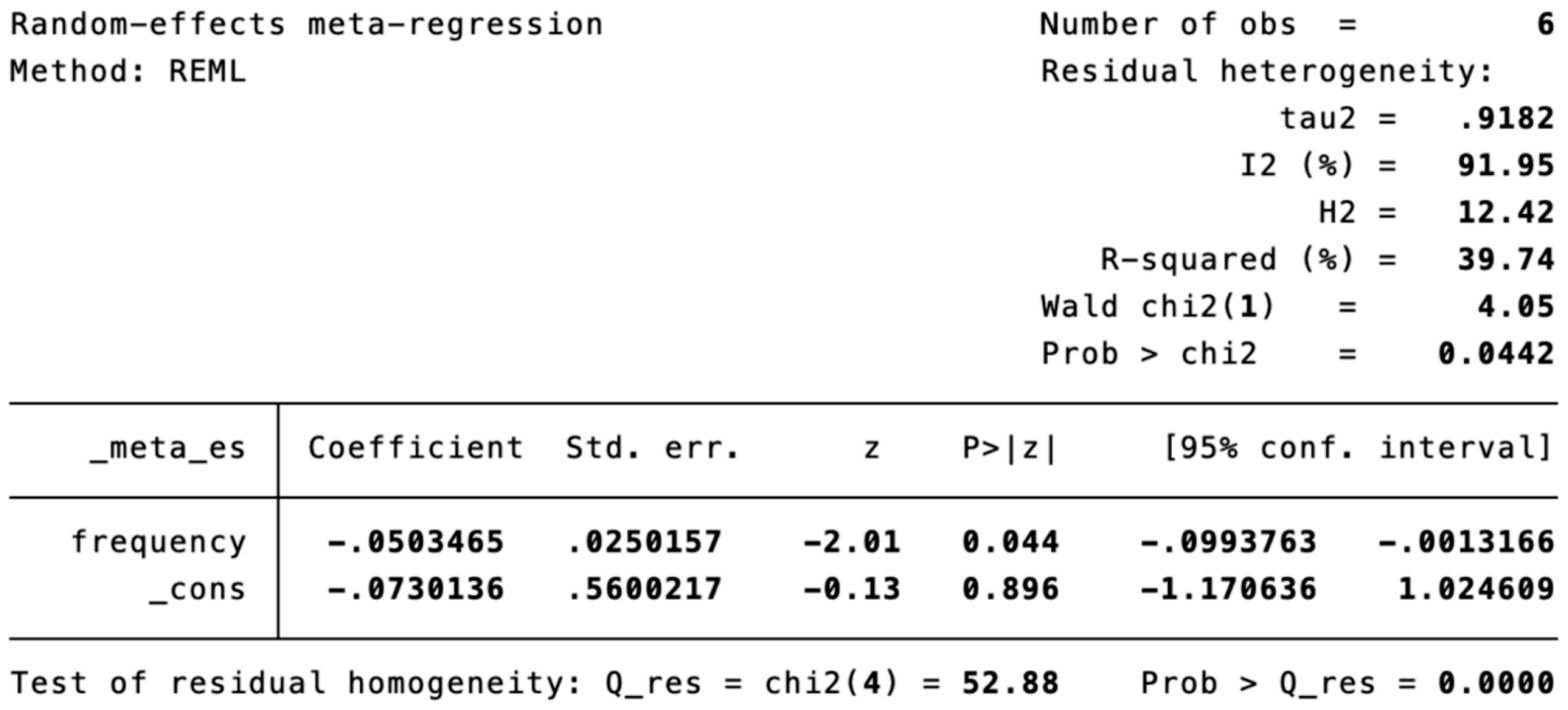
Meta-regression of treatment frequency.

Subgroup analysis (Figure 4) indicated that electroacupuncture was significantly more effective than simple acupuncture (MD = −6.12, *p* < 0.00001), but its effect did not reach statistical significance when compared to oral medication (MD = −1.76, *p* = 0.12). Furthermore, the difference between these subgroups was significant (*p* = 0.0003), suggesting that the type of intervention might be a source of heterogeneity. Subsequent sensitivity analysis based on funnel plots and Egger’s test were performed (Figures 5, 6). Meta-regression analyses were conducted after excluding studies with dual control groups. The results showed that neither the treatment duration nor the session length (Figures 7, 8), had a significant impact on the effect size (*p* > 0.05), with the regression models exhibiting low explanatory power (*R*^2^ approaching 0%). Additionally, since data on electroacupuncture treatment frequency could not be extracted from one study (21), a separate meta-regression analysis was performed after excluding this study (Figure 9). This analysis revealed a statistically significant negative effect of electroacupuncture frequency on the effect size, indicating that for each unit increase in frequency, the effect size decreased by approximately 0.05.

#### 3.4.2 Outcome: effective rate

**Figure 10:**
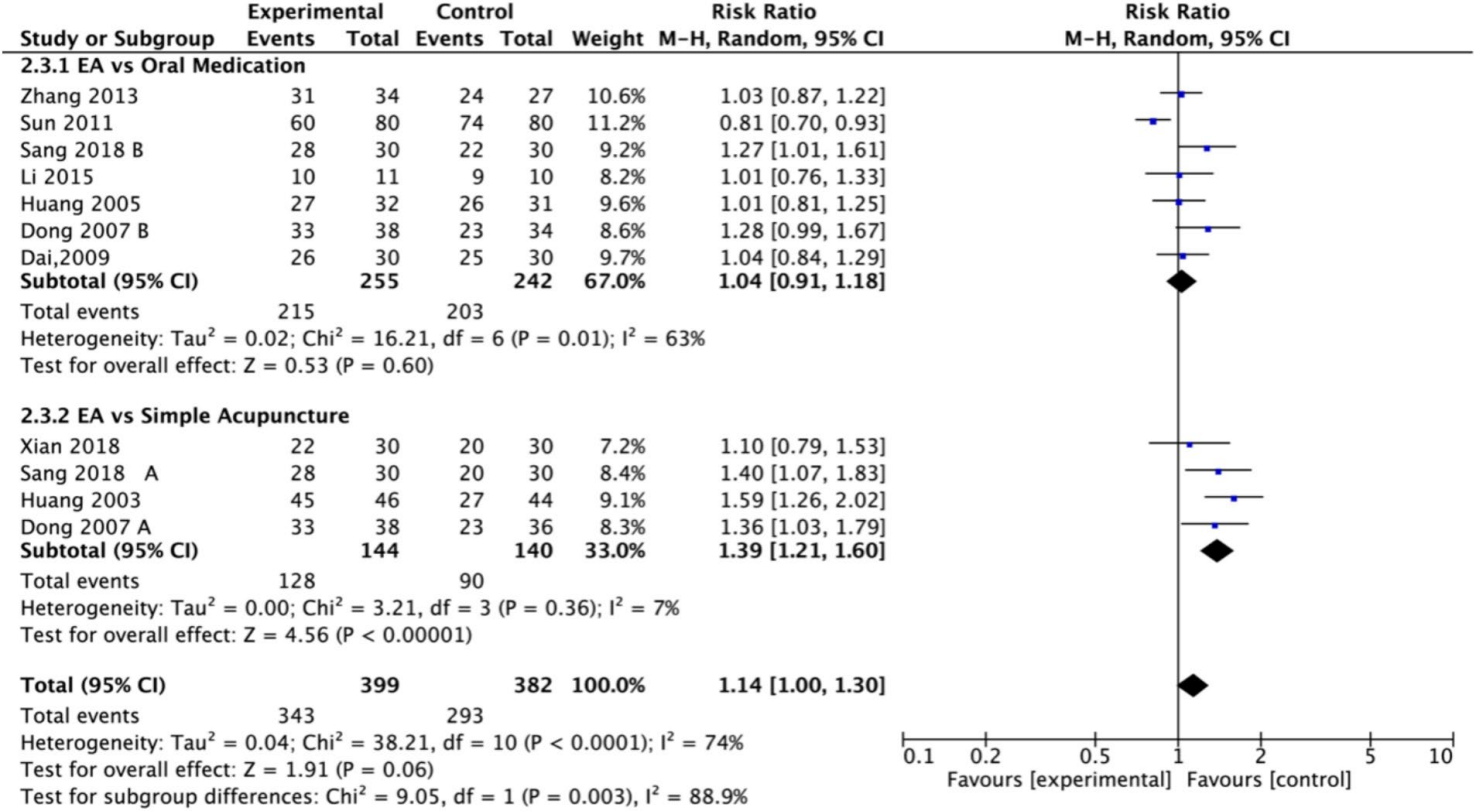
Forest plot of effective rate. EA, electroacupuncture; SD, standard deviation; M-H, Mantel–Haenszel; CI, confidence interval.

**Figure 11:**
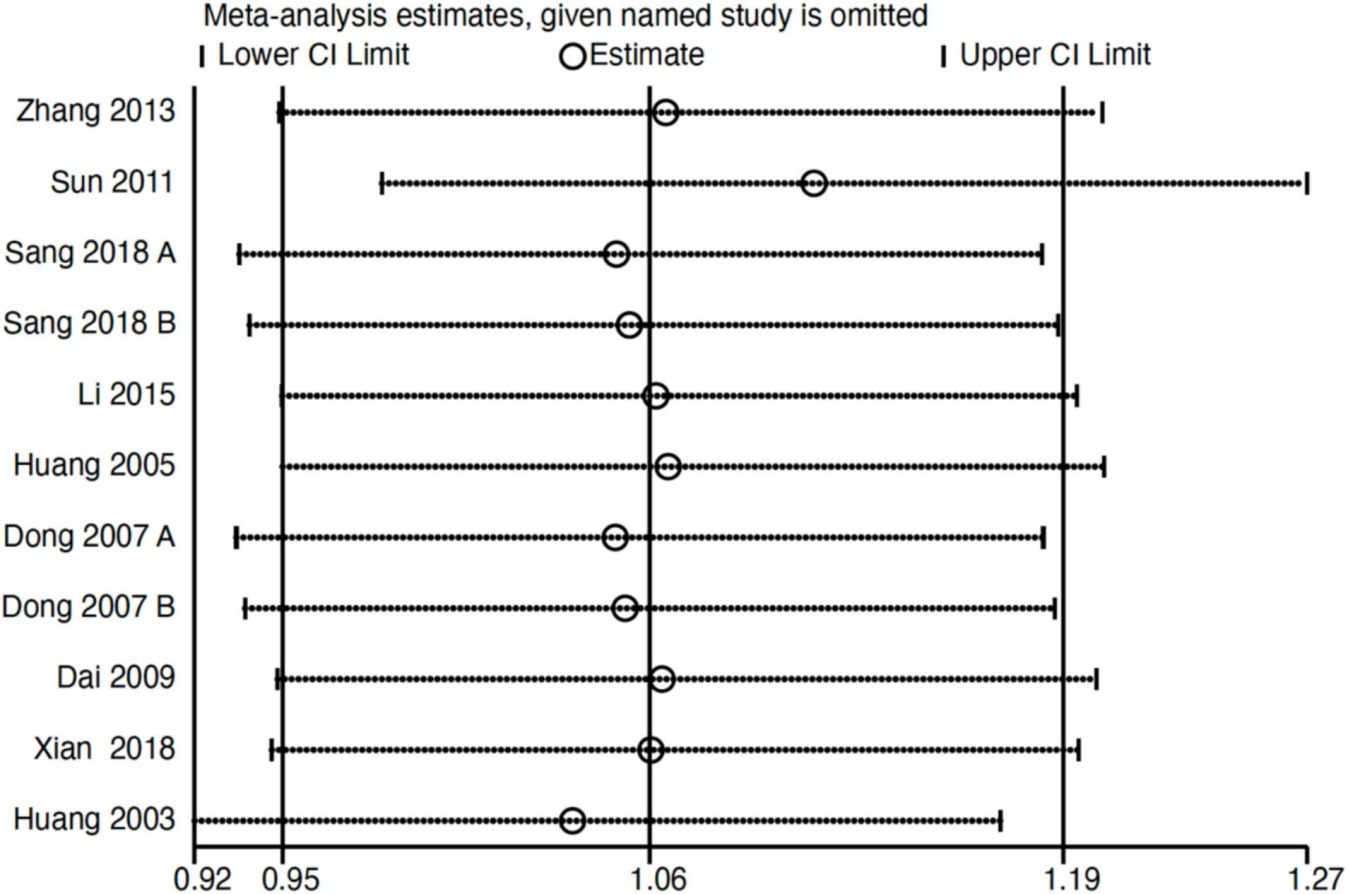
Sensitivity analysis of the effective rate. CI, confidence interval.

**Figure 12:**
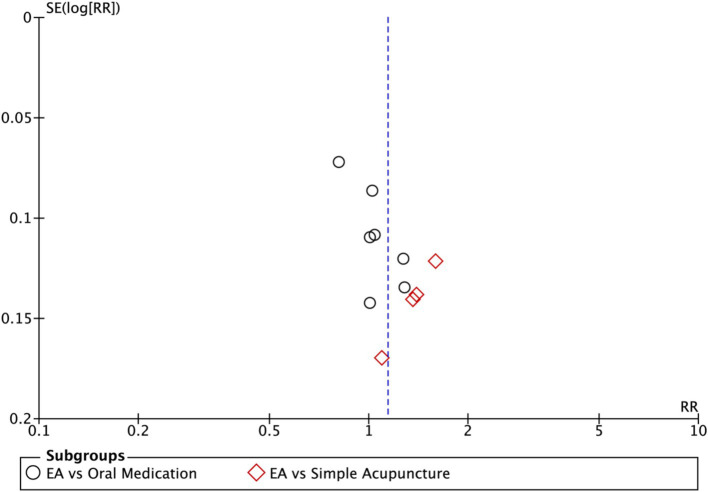
Funnel plot of effective rate. EA, electroacupuncture; RR, risk ratio; log [RR], logarithm of risk ratio.

**Figure 13:**
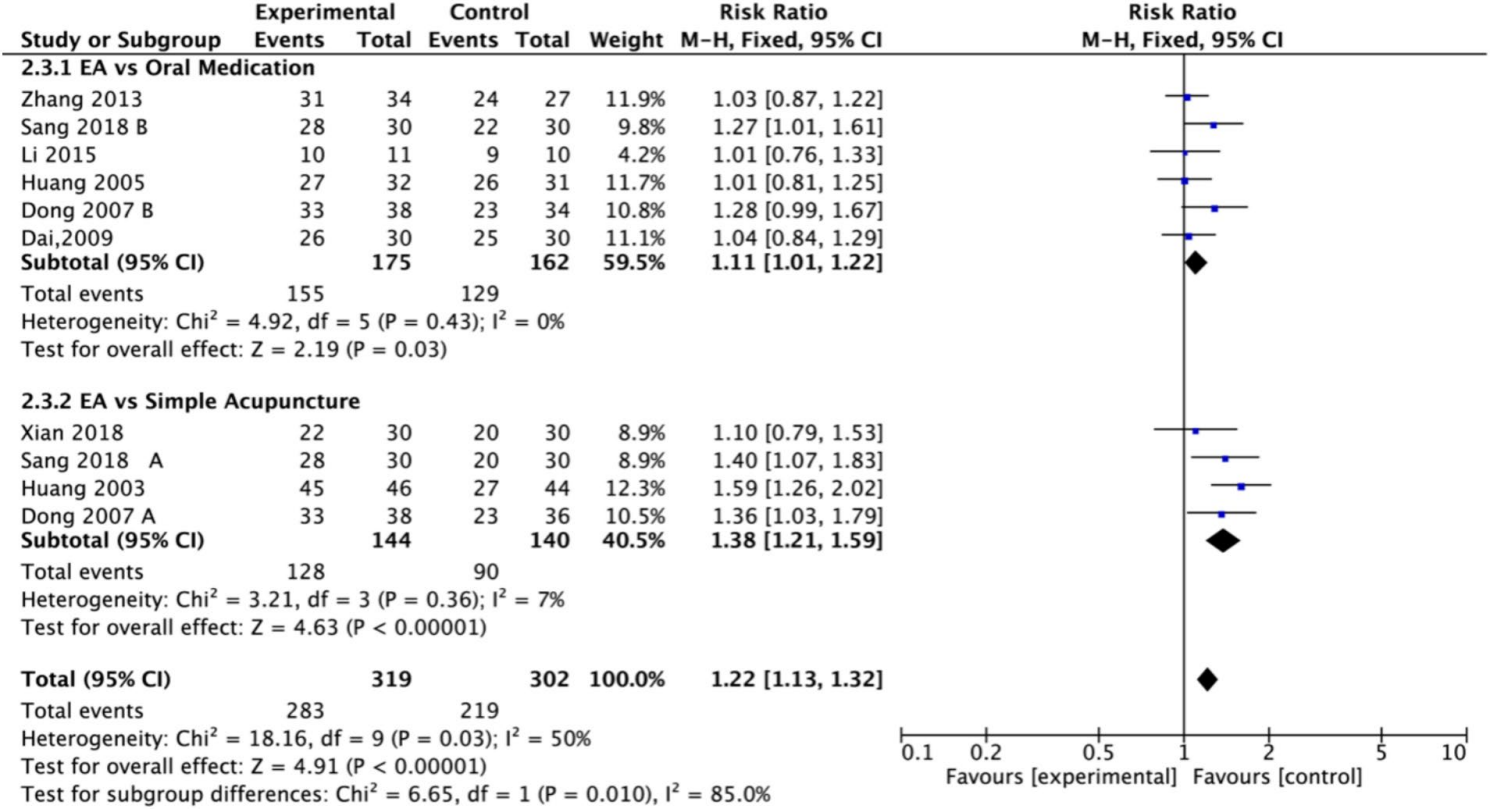
Forest plot of effective rate after excluding the study (26). EA, electroacupuncture; M-H, Mantel–Haenszel; CI, confidence interval.

**Figure 14:**
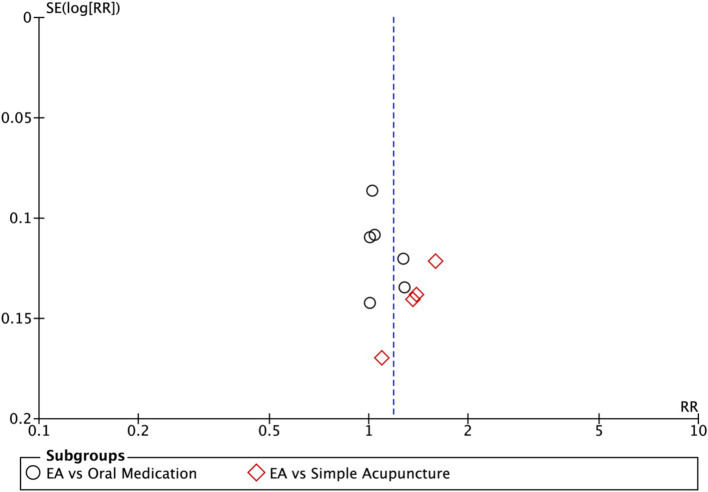
Funnel plot of effective rate after excluding the study (26). EA, electroacupuncture; RR, risk ratio; log [RR], logarithm of risk ratio.

Nine studies (*n* = 713) reporting clinical effective rates were included (17, 19, 20, 22–27). Subgroup analyses were performed stratified by control type. The overall random-effects model (*I*^2^ = 74%) demonstrated a significantly higher response rate in the electroacupuncture group compared to the control group (RR: 1.14, 95% CI: 1.00–1.30) (Figure 10). Due to substantial heterogeneity, subsequent sensitivity analyses based on funnel plots and Egger’s test were conducted (Figures 11, 12). As shown in Figure 13, excluding one study (26) from this comparison resulted in statistically significant changes in the results of the remaining studies (*I*^2^ decreased from 63 to 0% in the EA vs. OM subgroup). Accordingly, after excluding this study, a new forest plot and Egger’s test were generated (Figures 13, 14). The oral medication subgroup (6 studies; *I*^2^ = 0%), analyzed using a fixed-effects model, showed a significantly higher response rate in the electroacupuncture group (RR: 1.11, 95% CI: 1.01–1.22) (Figure 13). The simple acupuncture subgroup (4 studies; *I*^2^ = 7%), also analyzed with a fixed-effects model, confirmed a more pronounced advantage for the electroacupuncture group (RR: 1.38, 95% CI: 1.21–1.59) (Figure 13).

### 3.5 Secondary outcomes

#### 3.5.1 Outcome: self-rating depression scales

**Figure 15:**
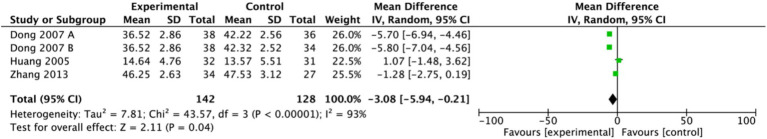
Forest plot of Self-Rating Depression Scale. Electroacupuncture vs. normal treatment or simple acupuncture. EA, electroacupuncture; SD, standard deviation; IV, inverse variance; CI, confidence interval.

A total of three studies (17, 19, 27) utilized self-rating depression scales. Lower scores on these scales indicate improvement in depressive symptoms. These studies involved a total of 104 participants in the experimental groups and 128 participants in the control groups. Due to substantial heterogeneity (*I*^2^ = 93%), a random-effects model was employed to evaluate this comparison. The results revealed that the EA group had significantly lower scores than the control group (MD: −3.08, 95% CI: −5.94 to −0.21) (Figure 15).

#### 3.5.2 Outcome: quality of life

**Figure 16:**
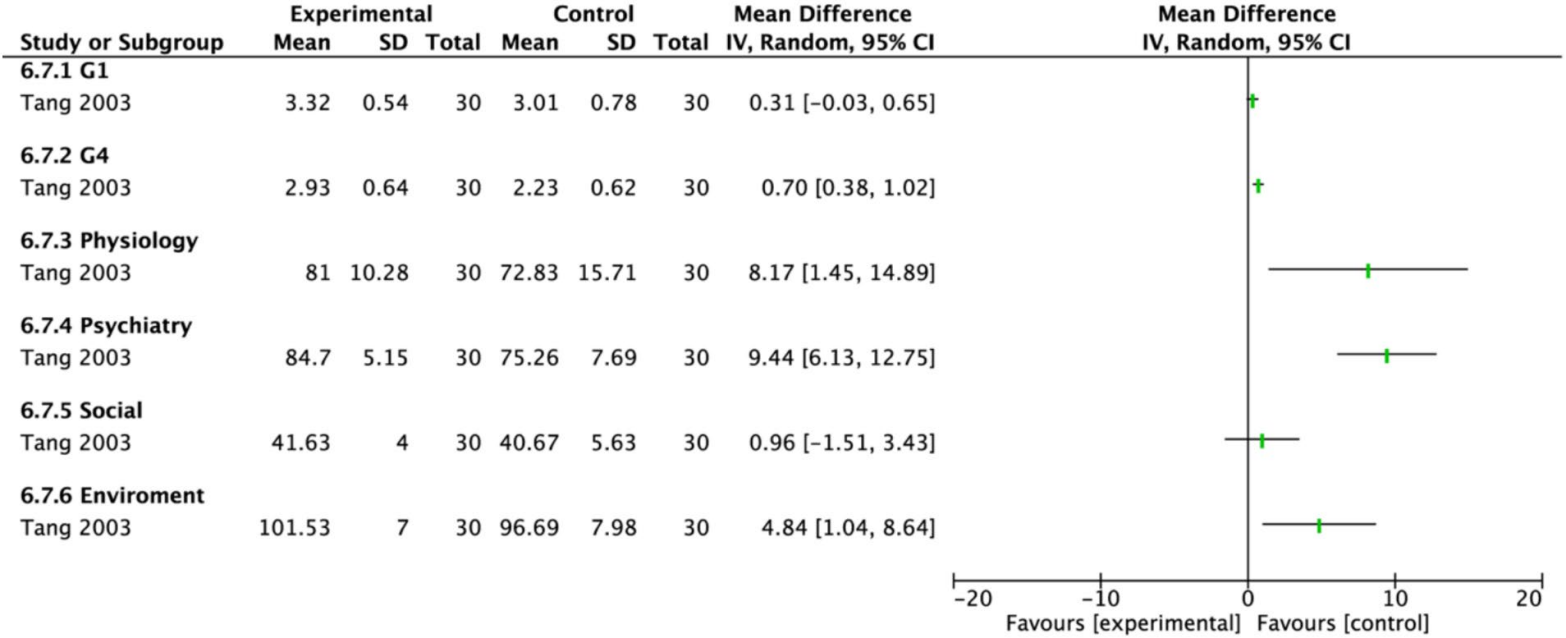
Forest plot of the World Health Organization Quality of Life-BREF. Electroacupuncture vs. No treatment. EA, electroacupuncture; SD, standard deviation; IV, inverse variance; CI, confidence interval.

**Figure 17:**
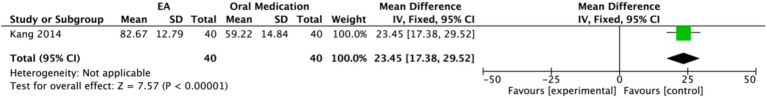
Forest plot of the Activity of Daily living Scale. Electroacupuncture vs. Oral Medication. EA, electroacupuncture; SD, standard deviation; IV, inverse variance; CI, confidence interval.

Two studies assessed quality of life using different scales. One study (18) involving 30 participants each in the EA group and the control group, used the abbreviated World Health Organization Quality of Life scale (WHOQOL-BREF) to evaluate multiple domains, including physical health, psychological health, social relationships, and environment. A random-effects model demonstrated a significant improvement in overall quality of life for the EA group compared to the untreated control group (MD = 1.85, 95% CI: 0.72 to 2.98, *p* = 0.001). Significant effects favoring EA were observed in the G4, physical health, psychological health, and environment domains (*p* < 0.05). Notably, psychological health showed the most pronounced improvement (MD: 9.44, 95% CI: 6.13 to 12.75, *p* < 0.00001), followed by physical health (MD: 8.17, 95% CI: 1.45 to 14.89, *p* = 0.02). The G1 domain showed a marginally significant effect (*p* = 0.07), while no statistically significant difference was found in the social relationships domain (*p* = 0.45) (Figure 16). In contrast, another study (21) used the Activities of Daily Living (ADL) scale for assessment (Figure 17). The results indicated that the ADL scores were significantly higher in the treatment group than in the control group (MD: 23.45, 95% CI: 17.38 to 29.52).

#### 3.5.3 Outcome: regional cerebral blood flow

**Figure 18:**
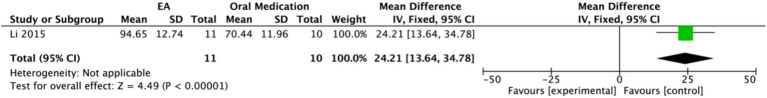
Forest plot of regional cerebral blood flow. Electroacupuncture vs. Oral Medication. EA, electroacupuncture; SD, standard deviation; IV, inverse variance; CI, confidence interval.

One study (23) assessed patients’ regional cerebral blood flow (rCBF). Higher rCBF values indicate better treatment outcomes. This study included 11 participants in the experimental group and 10 participants in the control group, and the data were evaluated using a fixed-effects model. The results showed that the EA group had significantly higher rCBF than the control group (MD: 24.21, 95% CI: 13.64 to 34.78) (Figure 18).

#### 3.5.4 Outcome: National Institutes of Health Stroke Scale (NIHSS)

**Figure 19:**
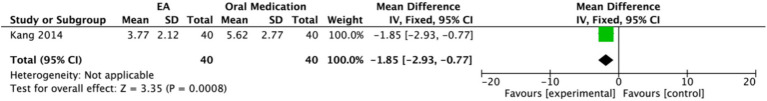
Forest plot of National Institutes of Health Stroke Scale. Electroacupuncture vs. Oral Medication. EA, electroacupuncture; SD, standard deviation; IV, inverse variance; CI, confidence interval.

One study (21) assessed patients’ neurological deficit scores using the NIHSS. Lower NIHSS scores indicate better treatment outcomes. This study included 40 participants each in the EA group and the control group, and the data were evaluated using a fixed-effects model. The results showed that the EA group had significantly lower NIHSS scores than the control group (MD: −1.85, 95% CI: −2.93 to −0.77) (Figure 19).

#### 3.5.5 Outcome: 5-hydroxytryptamine (5-HT)

**Figure 20:**
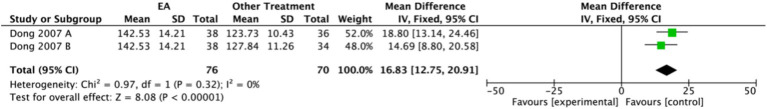
Forest plot of 5-Hydroxytryptamine. Electroacupuncture vs. Other Treatment. EA, electroacupuncture; SD, standard deviation; IV, inverse variance; CI, confidence interval.

One study (17) assessed patients’ plasma 5-HT levels. Higher plasma 5-HT levels are associated with improvement in depressive status. This study comprised an electroacupuncture penetrating acupuncture group (EA group, *n* = 38), and two control groups: a non-penetrating acupuncture group (*n* = 36) and a medication group (*n* = 34). The data were evaluated using a fixed-effects model. The results demonstrated that the EA group had significantly higher plasma 5-HT levels compared to the combined control groups (MD: 16.83, 95% CI: 12.75 to 20.91) (Figure 20).

#### 3.5.6 Outcome: stroke score

**Figure 21:**
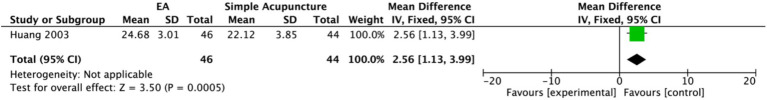
Forest plot of stroke score. Electroacupuncture vs. Simple Acupuncture. EA, electroacupuncture; SD, standard deviation; IV, inverse variance; CI, confidence interval.

One study (20) assessed patients’ stroke scores. Higher scores on this measure indicate better clinical outcomes. This study included 46 participants in the experimental group and 40 participants in the control group, and the data were evaluated using a fixed-effects model. The results demonstrated that the EA group had significantly higher stroke scores than the control group (MD: 2.56, 95% CI: 1.13 to 3.99) (Figure 21).

#### 3.5.7 Outcome: safety of EA

Among the 11 studies assessed, only one (26) reported adverse events. No adverse events were observed in the EA group. In contrast, adverse events such as dry mouth and sexual dysfunction were reported in the medication group. These findings suggest that EA is a safe treatment option.

#### 3.5.8 Outcome: overall evidence quality assessed by GRADE

**Figure 22:**
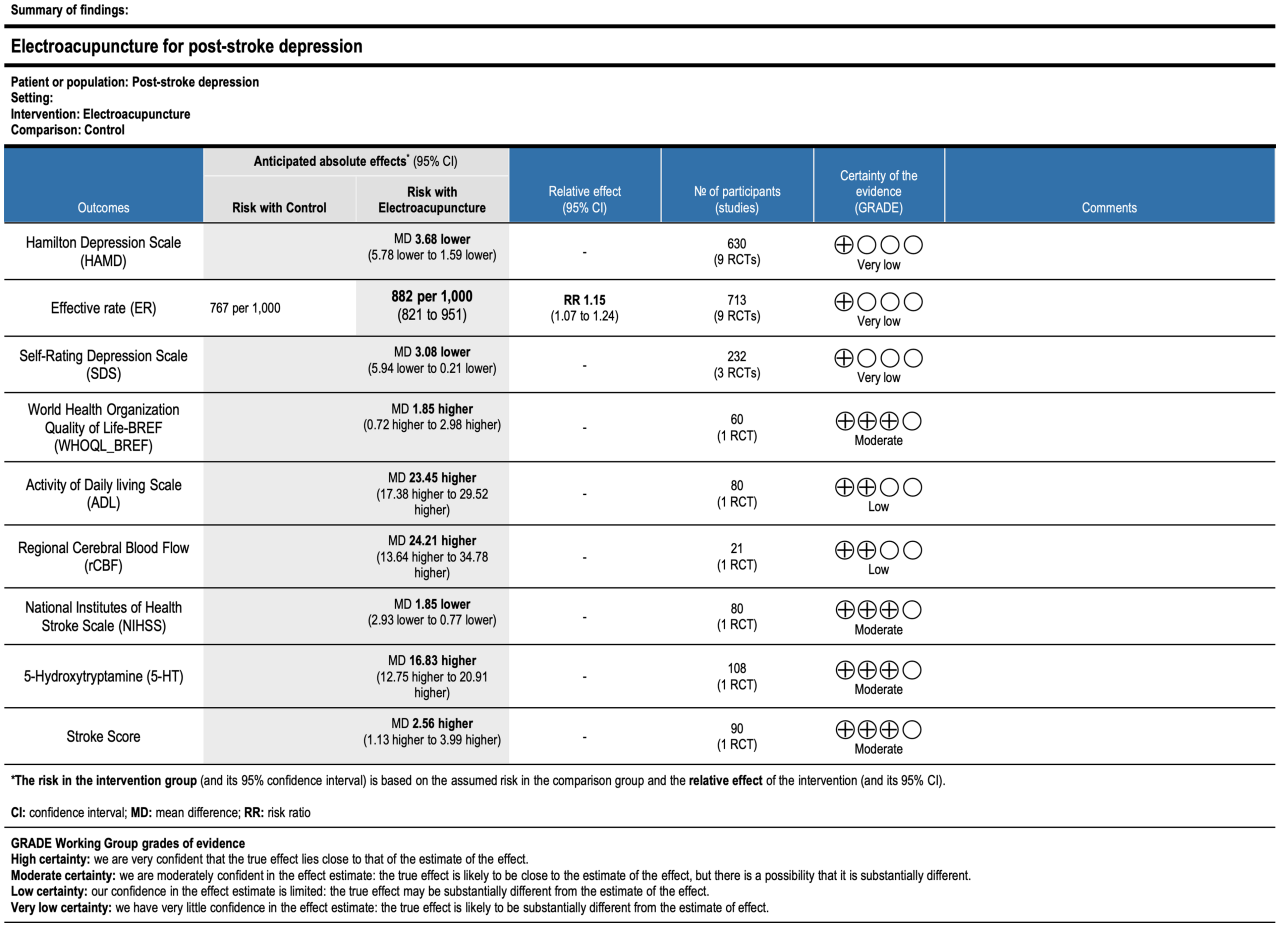
GRADE summary table of outcome indicator evidence quality. CI, confidence interval; MD, mean difference; RR, risk ratio.

The GRADE methodology was used to assess the overall quality of evidence for each outcome. The evidence quality was rated as “very low” for the HAMD, effective rate, and SDS. For ADL and rCBF, the evidence quality was rated as “low.” The evidence quality was rated as “moderate” for the NIHSS, 5-HT levels, and stroke scores. The evidence was downgraded for the following reasons: (1) publication bias was suggested by funnel plot asymmetry. (2) None of the included studies adequately described the methods for randomization, blinding of participants and personnel, or blinding of outcome assessment. (3) The majority of studies relied on subjective evaluation methods. (4) The number of included studies was small, and the methodological quality was generally low (Figure 22).

## 4 Discussion

This study analyzed data from 11 randomized controlled trials to investigate the efficacy of EA for PSD from multiple perspectives. The findings demonstrated that EA was superior to normal treatment and simple acupuncture alone in alleviating PSD. Furthermore, compared with pharmacotherapy or simple acupuncture alone, EA showed advantages in reducing depression scale scores, improving rCBF, enhancing quality of life (QoL), and increasing plasma 5-HT levels. These results suggest that EA treatment for PSD not only alleviates patient symptoms as measured by both subjective scales and objective indicators but also enhances QoL. Acupuncture has gained recognition in clinical practice worldwide. As a practical application of acupuncture, EA has also demonstrated clinical efficacy in treating depression and stroke (28). Traditional Chinese Medicine (TCM) posits that EA can regulate the flow of Qi within the meridians, thereby ameliorating stroke symptoms. Furthermore, this anti-inflammatory mechanism provides a plausible biological basis for the specific outcome measures that improved in our analysis. For instance, the observed increase in plasma 5-HT levels may be directly linked to the attenuation of neuroinflammation. Chronic inflammation is known to disrupt serotonergic pathways, partly by upregulating enzymes that divert the precursor tryptophan away from 5-HT synthesis. By inhibiting pro-inflammatory signaling cascades like the TLR4/NF-κB pathway, EA may help normalize tryptophan metabolism, thereby increasing the availability of 5-HT (29). Similarly, post-stroke neuroinflammation contributes to endothelial dysfunction and impairs cerebral microcirculation. The anti-inflammatory action of EA may help restore vascular integrity and function, leading to improved perfusion in affected brain regions. Previous evidence indicates that EA can mitigate brain damage following stroke and improve other stroke complications, such as disability and cognitive impairment (30, 31). Given the multifaceted nature of stroke, patients with PSD frequently experience concurrent complications, including hemiplegia, aphasia, and urinary retention. All these complications can co-occur, complicating the treatment process. Therefore, concurrent treatment of both stroke and depression is crucial. Previous research has shown that EA promotes recovery from cerebral ischemia and addresses stroke complications (32–34). Moreover, numerous systematic reviews and meta-analyses have highlighted the benefits of acupuncture for PSD. For instance, one study evaluate the differences between EA and sham EA in treating depression. The results revealed significantly greater improvement in HAMD scores in the EA group compared to the sham EA group post-treatment (*p* < 0.001). However, this study relied solely on subjective scales for assessment, neglecting objective indicators such as plasma 5-HT levels (35). Additionally, a meta-analysis conducted in China evaluated the therapeutic effect of eye acupuncture combined with other normal treatments on patients with PSD (36). This study analyzed the effective rate (RR: 1.22, 95% CI: 1.12 to1.33), HAMD scores (MD: 3.22, 95% CI: 3.04 to 3.39), and SDS scores (MD: 3.73, 95% CI: 3.63 to 3.83), suggesting a potential advantage of eye acupuncture for PSD (36). Notably, our study primarily evaluated PSD treatment efficacy for PSD by assessing HAMD scores (MD = −3.68, 95% CI: −5.78 to −1.59, *p* = 0.0006) (Figure 3) and the effective rate (RR: 1.14, 95% CI: 1.00 to 1.30) (Figure 10). A particularly important finding from our subgroup analysis was the lack of a statistically significant difference in HAMD score reduction between the EA and oral medication groups. While this result does not demonstrate the superiority of EA, it strongly suggests that EA may be non-inferior to conventional antidepressants in efficacy. This is a clinically significant outcome, especially when considering the broader therapeutic profile. Our review also noted that the included studies reported fewer adverse events, such as dry mouth and sexual dysfunction, in the EA groups compared to the medication groups. Therefore, for patients who cannot tolerate or do not respond to pharmacotherapy, EA may present a valuable alternative with a more favorable profile. It is also crucial to acknowledge that this finding may be attributed to the limited statistical power from the small number of trials included in this specific analysis. This underscores the urgent need for larger, more robust RCTs directly comparing EA with standard antidepressants to definitively establish its relative efficacy in the clinical management of PSD. Furthermore, we analyzed other detailed aspects, including objective indicators such as rCBF and plasma 5-HT levels, as well as QoL metrics. On one hand, QoL represents a crucial aspect of holistic patient management. On the other hand, objective measures like rCBF and plasma 5-HT levels are valuable for evaluating diagnosis and treatment. In summary, our findings indicate that EA, which combines acupuncture with electrical stimulation, leverages the strengths of both modalities and demonstrates superior therapeutic efficacy compared to each modality used alone.

As an intervention requiring penetration of the skin and muscle tissue, the safety of EA is paramount. A previous systematic review (37) reported that during EA combined with magnetic stimulation for treatment-resistant depression, adverse events were primarily localized pain and mild nephritis. Among the 11 trials included in this study, only one (26) mentioned and reported relevant adverse reactions. Moreover, all reported adverse events occurred in the control group, indicating favorable safety of EA (38–40).

Overall, this study possesses the following methodological strengths. First, to our knowledge, this is the first systematic review and meta-analysis focusing exclusively on the efficacy and safety of EA monotherapy for PSD. Second, compared with previous studies, we incorporated more recent trials, clarifying the superior efficacy of EA over both normal treatment and simple acupuncture alone. Third, we independently evaluated the effects and safety of both EA monotherapy and combination therapies. Finally, regarding clinical implications, this meta-analysis confirms that EA is applicable for depression management across all stroke phases and may exhibit superior efficacy compared to normal baseline treatment and simple acupuncture alone. However, several limitations warrant acknowledgment. First, all included studies were conducted in mainland China, and the small sample sizes across RCTs restrict the generalizability of findings beyond this region. Multicenter, large-scale RCTs are warranted to facilitate global translation of these findings. Second, some included studies have a wide range of disease durations, unclear descriptions of baseline treatment methods and high risk of bias in randomization procedures. Third, while existing evidence suggests a favorable safety profile for EA, comprehensive safety data specifically for the PSD population remain limited. Future RCTs should rigorously report key methodological details, including blinding, random sequence generation, and allocation concealment.

In conclusion, while most subjective outcomes (e.g., HAMD/SDS scores and QoL) demonstrated statistical significance in the current evaluation system, reliability may be compromised by heterogeneity in assessment protocols across studies. Prospective studies implementing standardized assessment procedures are needed for validation. Concurrently, the limited reporting of adverse events and post-treatment follow-up data impedes comprehensive safety evaluation and obscures long-term effects, potentially diminishing the clinical utility of these findings.

## 5 Conclusion

EA demonstrates superior efficacy compared to normal treatment or simple acupuncture alone in managing PSD. EA may represent an effective therapeutic approach for PSD and a viable component of comprehensive PSD management strategies. However, due to the high overall risk of bias and substantial loss to follow-up in long-term RCTs, more rigorous studies are required to confirm these findings, as current clinical evidence remains inconclusive.

## Data Availability

The original contributions presented in the study are included in the article/supplementary material, further inquiries can be directed to the corresponding author.
